# Fast Readout Architectures for Large Arrays of Digital Pixels: Examples and Applications

**DOI:** 10.1155/2014/523429

**Published:** 2014-03-19

**Authors:** A. Gabrielli

**Affiliations:** Physics and Astronomy Department and INFN, University of Bologna, Viale Berti Pichat 6/2, I 40127 Bologna, Italy

## Abstract

Modern pixel detectors, particularly those designed and constructed for applications and experiments for high-energy physics, are commonly built implementing general readout architectures, not specifically optimized in terms of speed. High-energy physics experiments use bidimensional matrices of sensitive elements located on a silicon die. Sensors are read out via other integrated circuits bump bonded over the sensor dies. The speed of the readout electronics can significantly increase the overall performance of the system, and so here novel forms of readout architectures are studied and described. These circuits have been investigated in terms of speed and are particularly suited for large monolithic, low-pitch pixel detectors. The idea is to have a small simple structure that may be expanded to fit large matrices without affecting the layout complexity of the chip, while maintaining a reasonably high readout speed. The solutions might be applied to devices for applications not only in physics but also to general-purpose pixel detectors whenever online fast data sparsification is required. The paper presents also simulations on the efficiencies of the systems as proof of concept for the proposed ideas.

## 1. Introduction

Pixel device systems are always under investigation for applications in future high-energy physics (HEP) experiments or in upgrade of current colliders, like the Super-Large Hadron Collider (SLHC) [1, 2] that will be the natural evolution of the current LHC [3–5] experiment at CERN of Geneva. The continuous increase of luminosity [6, 7] and, consequently, the amount of data to be read out forces the pixel detectors [8, 9] into designing on-chip fast readout electronics. This in fact can significantly increase the overall performance of the system. Innovative solutions are deemed particularly useful for large, low-pitch pixel circuits that implement huge pixel connectivity via very large control and data buses. In addition, routing between the sensors can produce false hits, due to induced noise. Hence this paper proposes to use only interpixel global wires despite point-to-point wires from the border of the matrix to single pixels or groups of pixels (global wires only). The approach sets out to simplify interpixel routing by moving registers and sparsification [10–12] logic outside the matrix within a less congested and less critical area. Here, sparsification process refers to the identification of the pixels to be read out, within the entire matrix of pixels, and to the data reduction due to the readout efficiency [13]. In other words, sparsification might be traduced as fast and dedicated readout logic focused only to those pixels that have been crossed by ionized particles, that is, hits. The circuits that carry out sparsification are called sparsifiers and since only the hit pixels are read, the entire readout process takes the minimum time with respect to the amount of pixels in the matrix that might be read out, and this is a data and time reduction process. The concept is to read out the columns of pixels one at a time so that all hits belonging to the same column are detected in parallel and independently of the number of the rows and columns. The pixels of the investigated column—active column—that have not been hit are consequently ignored. In principle, the entire readout phase takes as many clock periods as the number of columns that has hits. It follows therefore that the readout speed might be significantly reduced with respect to that of recently used pixel detectors where a token-like technique is implemented [14, 15]. Additionally, as in normal practice, each matrix sweep cycle is associated with a time stamp to reconstruct the hits according to the time they were produced. For this the columns activated within the same event are frozen until they are read out. As the pixel logic does not require any internal register, time stamps can be saved outside the matrix of pixels within a less congested area. In conclusion, the solution features interpixel wiring independent of the size of the matrix, since there are no point-to-point wires and all lines are global. This readout approach can easily be extended to any size matrix, as it is independent of the number of pixels. This paper proposes readout architectures for pixel devices where traditional, insufficiently fast structures, such as token-passing techniques, cannot fulfill speed requirements. For low-occupancy devices, this technique should be considered for its simplicity as it could also be used in future improvements in the electronics of physics experiments.

## 2. Electronics for Particle Colliders

A typical (silicon) solid-state detector [16–18] is composed by the following.

**Figure 1:**
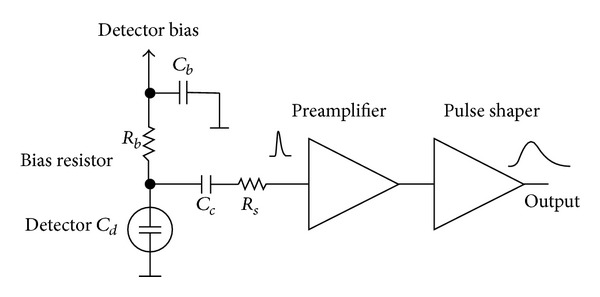
Typical detector front-end circuit.

- A sensor: this is the sensitive part of the detector. It is commonly a capacitive element to collect the charge collected in the silicon substrate. It translates the charge into a voltage signal (for minimum ionizing particles the most probable charge deposition in a 300 *μ*m thick silicon detector is about 3.5 fC (22000 electrons). The sensor is typically implemented as a reverse-biased p-n junction, which forms a region depleted of mobile charge carriers and sets up an electric field that sweeps the charge generated by radiation and diffusing in the substrate.
- An analog front-end; this is the analog electronics directly connected to the sensor, its task is to amplify, adapt, and discriminate the sensor signal with a voltage threshold. Keeping the front-end noise low is a critical issue to improve the energy resolution, which depends on the collected charge, and to allow a low detection threshold. For certain energy values, particles are more reluctant to ionize and release less charge; the electronics ENC (Equivalent Noise Charge) [19, 20] should be below this value. A scheme of a typical front-end circuit is shown in Figure 1.
- A latch: this is the memory element that keeps track of a threshold crossing. It is reset after the channel has been read out. The longer it takes to read and reset the latch, the longer the sensor is “blind” to new incoming particles. In fact, while the sensor has to be read out, it is kept frozen and, consequently, insensible to new particle crossings.
- A readout circuit: this is the electronics appointed to extract the hit information from each pixel latch. It can be implemented in very different ways depending on the optimization targets. This is also the element on which we focused our work.

Also monolithic devices use the same block architecture but share the same silicon layer for sensing and reading the hits [21–23].

Readout electronics used in modern pixel radiation detectors that have been designed and constructed for HEP are commonly built using general architectures that require a serial scan of the matrix. Experiments use bidimensional matrices of sensitive elements located on a silicon die. The speed of the readout electronics can significantly increase the overall performance of the system, and so this paper analyzes a novel form of readout architecture for pixel devices. These circuits have been investigated in terms of speed and might suit big monolithic, low-pitch pixel matrices. It is here that a simple structure expandable to larger matrices is proposed. This solution might be applied to systems for applications in physics and to more general-purpose pixel detectors whenever online fast data sparsification is required.

A particle collider is composed of many nested detectors for different purposes. Basically, the innermost layers of detection are aimed at tracking the particles or the secondary recoils of particles, which come out immediately after the collision of two bunches of primary particles. These bunches are circularly accelerated close to the speed of light in clockwise and counter clockwise directions, before the interaction is being induced. The inner layers of detections are primarily based on pixel devices located over a cylindrical surface of a supporting barrel.

**Figure 2:**
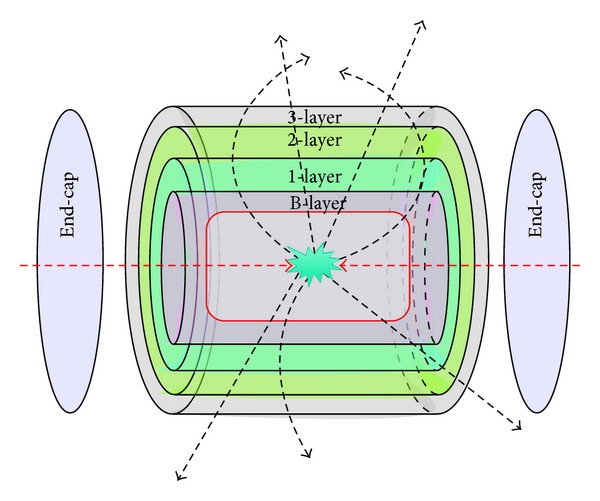
Sketch of the nested layer of the pixel detector of ATLAS.

In recent years particle colliders, such as the Large Hadron Collider (LHC) at CERN in Geneva, have reduced the time interval between consecutive bunch crossings and the electronics used in the data acquisition chains of the experiments that have used advanced submicron technologies available in the market. As an example, Figure 2 shows a sketch of the pixel detector of the main experiment at CERN [24–29], that is, ATLAS, with its 4 nested pixel layers, namely, B, 1, 2, and 3. In addition, in these experiments innermost detectors are generally composed of solid-state devices such as arrays of silicon pixels. The choice relies on the small pixel dimension that allows for a very high spatial resolution, of the order of tens of *μ*m [30–32]. These inner detectors, basically, just detect the passage, or not passage, of ionizing particles through the matter and, for this reason, these apparatus are called trackers [33, 34]. Trackers are those parts of a more complex particle detector dedicated to the identification of real physics events from the background noise and the specific devices that sense the particles just after their appearance are the pixel detectors.

As most ionizing particles produced by the collision event leave their charge in a group of pixels read by specific readout electronics, the latter measures the released charge. High-energy physics experiments (HEPE) have been equipped with front-end electronics composed of integrated circuits using CMOS technologies compatible with the readout speeds and working frequencies [35–37].

In particular, many experiments that use silicon pixel detectors have implemented sensors built with custom integrated circuits. The number of pixels in the basic readout unit, which may be a chip or module, is of the order of some thousands (2880 for ATLAS [24–26]), 4160 for CMS [27, 28], 8192 for ALICE [29–31]). Of course, the number of pixels in a system is vastly larger. These pixels are arranged in matrices of 256 rows by 32 columns for ALICE, 80 rows by 52 columns for CMS, and 160 rows by 18 columns for ATLAS. The arrays of pixels are located on a first chip bump bonded on a second silicon die that houses the readout electronics [34–36]. This means the entire microelectronics design consists of several components implemented on different silicon integrated circuits even though these might be merged into one single component. In this paper a hit corresponds to a fired pixel as a consequence of an injected charge whatever its origin.

**Figure 3:**
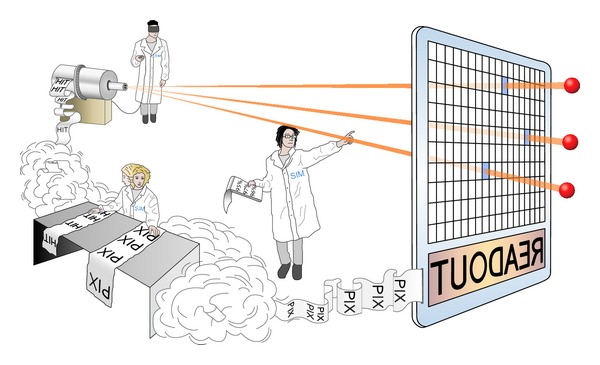
Hit identification concept.

The hit information is associated with the spatial coordinates and the time when the pixel was hit.  Figure 3 shows a pictorial view of a hit creation from particle passage through a detector. The readout system is the circuit that reconstructs the time and space information of the individual hits, that is, when a hit has occurred and in what position within the matrix of pixels. In principle, the readout data can be held within or outside the pixel circuit. For this reason in many HEPE some registers are inserted within the pixels; however, here it has been chosen to design a very simple pixel and move the readout electronics outside the matrix of sensors. This is deemed particularly useful for small (of the order of 100 *μ*m^2^) active pixel sensors such as those exploited for future colliders [32–34, 38, 39]. This also allows for designing and exploiting larger matrices of pixels.

The readout architecture also depends on how the pixels are designed, arranged, and connected. In addition to the readout technique viewpoint, the greater the number of wires routed among rows and columns of pixels, the larger the pixel pitch and hence the lower the detector spatial resolution. In addition, routing between the sensors can produce false hits due to induced noise. As here spatial resolution together with readout speed have been investigated, the paper presents a new and faster approach for reading out the hits over the matrix of pixels wherever they are located—an approach that would particularly suit monolithic large-sized low-pitch matrices. The approach has been already implemented on some prototypes of Monolithic Active Pixel Sensors (MAPS) [37, 40–49] that were investigated for future trackers of particle colliders [50].

## 3. Typical Techniques: CCD-Like Readout

**Figure 4:**
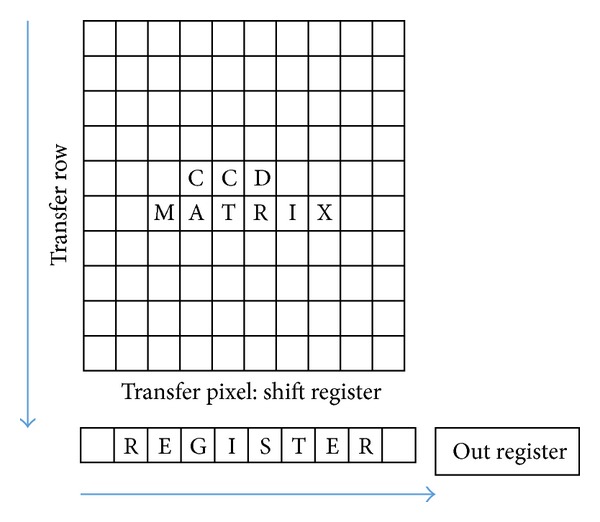
CCD readout.

A charge-coupled device (CCD) [51] is a well-known device normally implemented into an integrated circuit. A CCD is mainly composed of a silicon surface forming light sensitive elements, also called pixels, primarily used for imaging applications [52]. The light incident on the CCD surface creates charge that must be read by electronic circuits. CCDs are mainly used into digital cameras or electronic microscopes like Scanning Electron Microscopes (SEM) and Transmission Electron Microscopes (TEM) [53, 54] and, for some niche applications, also in physics experiments and more general high-end scientific applications [55]. However, the readout electronics typically used for CCDs is independent of the application and, consequently, reads out the CCD information whatever the origin of the charge. In other words a CCD is a bidimensional array of pixels collecting photons or ionizing particles, which leave a given amount of charge in the substrate. We can summarize that, for the detector viewpoint, the main difference between ionizing particles and photons, particularly for high-energy particles, is that ionizing particles deposits a bigger amount of charge with respect to photons. In the visible spectrum in fact, photons turns into individual electrons in the range of a few eV of energy. Thus, for photons each pixel behaves like a bucket exposed for a given amount of time to a “rain of light.” Eventually, the pixels fill up with a varying amount of charge, and the CCD is then read sequentially one pixel—bucket—at a time. This process is carried out when each pixel of a column in the array pours charge into the adjacent empty cell.  Figure 4 shows the charge transfer from top to bottom along the columns and, eventually, from left to right towards an output register. Hence, step-by-step the pixels in a column transfer their charge up to a final pixel, where the readout electronics of the CCD reads out this last pixel. The charge is then converted into a number that can be understood and interpreted.

Electrons of the stored charge are shifted in two directions on a CCD. All columns work in parallel by shifting charge from top to bottom. Then serial shift is performed from left to right and directs the electron packets to the measurement electronics.

Generally, CCDs are built with multiple amplifiers at each corner and can thus be read out faster but, in any case, the CCD is originally a sequential element.  Figure 4 shows schematically CCD readout.

## 4. Typical Techniques: Token Ring Readout

The concept of a token-passing technique is copied from a type of computer network, where all the computers are organized in a circular architecture. IBM studied the token ring architecture in the 1980s [56] and the specifications were defined in the IEEE 802.5 [57] standard. IBM designed token ring to connect different computer types, like personal, home computers and mainframes. A token, commonly composed of a pattern or bits, travels around the circle from one computer to another one. When a computer needs to send a message, it takes the control of the network by catching the token, rewriting it by attaching or updating a new set of bits, and then by releasing the token. Then, the token continues its trip around the network. While one of the computers is holding the token, the others are not allowed to send messages. Now, if we imagine replacing the computers with pixels, or column of pixels in a matrix, and the token with the hit information to be read out, then the token-passing concept can be easily adapted and used also in pixel readout architectures.

**Figure 5:**
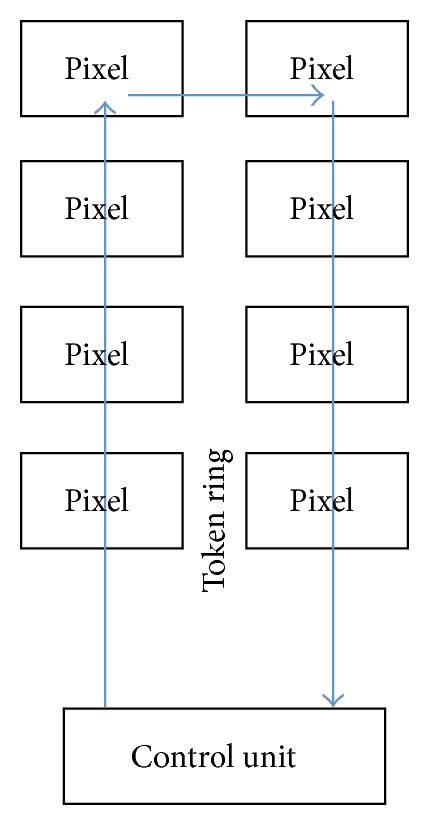
Token ring readout.

One of the first applications in high-energy physics where the token-passing technique was exploited to read out a pixel detector was the BTeV [58, 59] experiment at Fermilab (http://www.fnal.gov/), USA. Since BTeV planned to use a pixel detector as part of the trigger system, the most important characteristic was speed. The primary goal was then to achieve a readout rate close to five hits per beam crossing. The pixel readout in the chip [60, 61] was chronologically organized by time stamp. Within the column the readout was organized by taking into account the pixel's physical location. A pixel grouping technique with two levels of token-passing hierarchy provided a simple and very fast way of locating hit pixels during the readout cycle. The number of readout controllers, the number of time stamp registers, the clock frequency of the communication channels, and the depth of the on chip buffers were critical in the design of the architecture.  Figure 5 schematically shows how a token ring, token-passing technique, is achieved. As said, along a physical ring a token is passed from one pixel to an adjacent one. The control unit has the control of the system but each individual pixel can hold the token to attach further information. The advantage of this technique is the reduced complexity of the layout, which is independent of the size of the matrix of pixels. By contrast, if the ring breaks, the entire matrix addressing is lost. In addition, the logic is substantially sequential, leading to a low-speed readout. This technique is a reasonable solution, where a fast readout is not necessarily requested and a human intervention is feasible to fix communication problems. This is why computer networks were the natural application for a token-passing readout technique.

## 5. An Innovative Readout Technique

**Figure 6:**
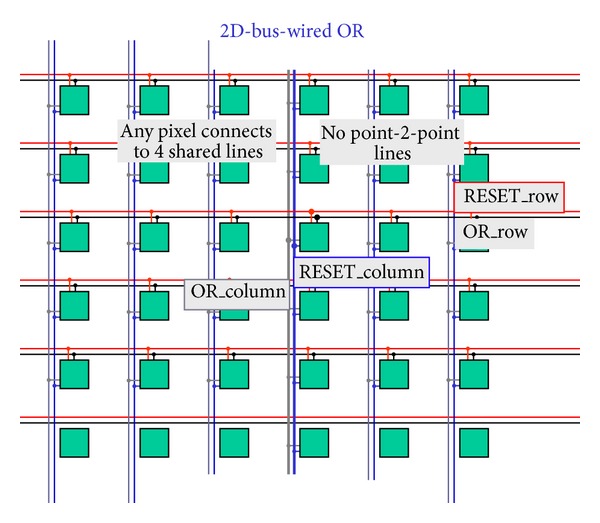
The wired-or logic.

**Figure 7:**
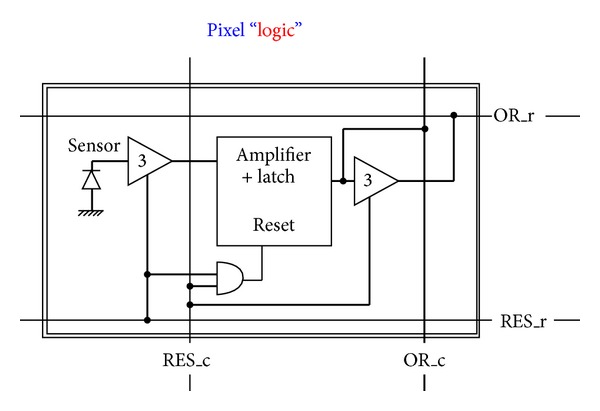
The 4-wire pixel logic.

The idea is to use only interpixel global wires and not point-to-point wires from the border of the matrix to single pixels or groups of pixels [62].  Figure 6 shows a diagram of the pixels that can be driven and read out via global wires only. Wire density and pixel pitch do not depend on the number of pixels (size of the matrix). The approach sets out to simplify interpixel routing by moving registers and sparsification logic outside the matrix within a less congested and less critical area.  Figure 7 shows a sketch of the proposed 4-wire in-pixel logic whatever the design of the sensor. Each pixel simply connects to the 4 wires that are then shared over the entire matrix. Consider the following in detail.

- OR_r is a 3-state buffered horizontal output wire to read the pixel status. When the buffer is enabled through the RES_c vertical line, pixel output is read via the OR_r wire. This line is shared with all pixels in the same row by creating a wired-or condition. As only one pixel at a time is allowed to be read, the OR_r coincides with the pixel's output value.
- RES_r is a horizontal input wire to freeze the pixel by disconnecting it from the sensor. Moreover if RES_r is asserted along with the RES_c line, it resets the pixel. Also this line is shared with all pixels in the same row.
- OR_c is a vertical output line that is always connected to pixel output. This is shared with all pixels in the column by creating a wired-or condition. If at least one pixel of the column is on, the wire is obviously on too, independently of the number of hits and their location.
- RES_c is a vertical input line to enable the connection to the sensor via a 3-state buffer. As described below, it is used to mask an entire column of pixels. Again, if used with the RES_r, it resets the pixel.

**Figure 8:**
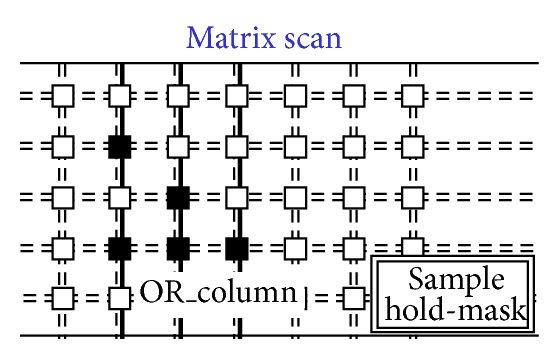
Columns and rows of the hits.

**Figure 9:**
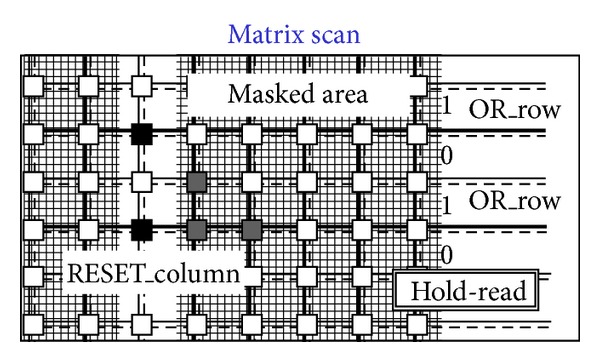
Readout starts for the first enabled column.

**Figure 10:**
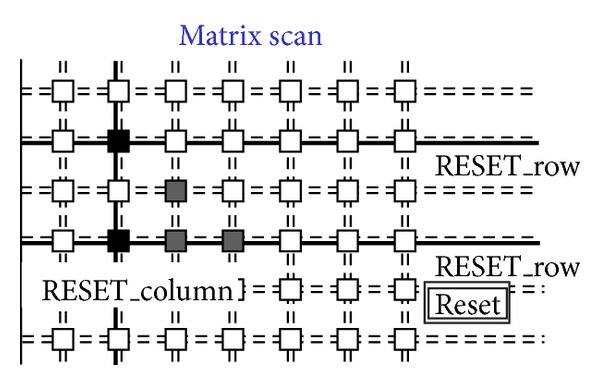
First reset on the enabled column.

**Figure 11:**
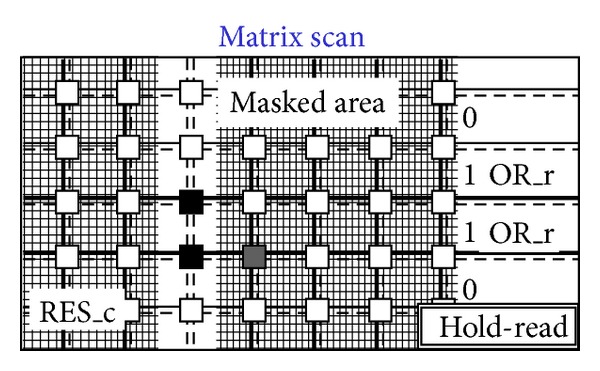
Second enabled column.

**Figure 12:**
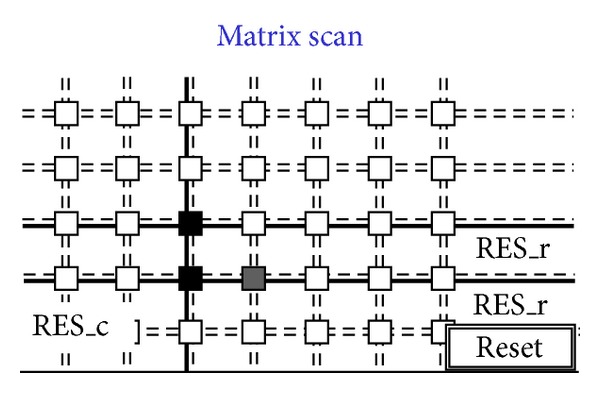
Second reset on the columns.

**Figure 13:**
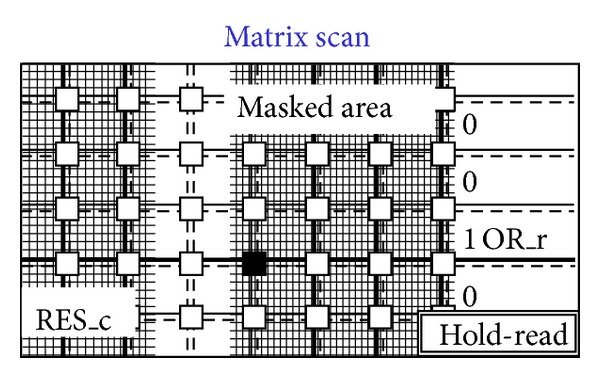
Last selected column.

**Figure 14:**
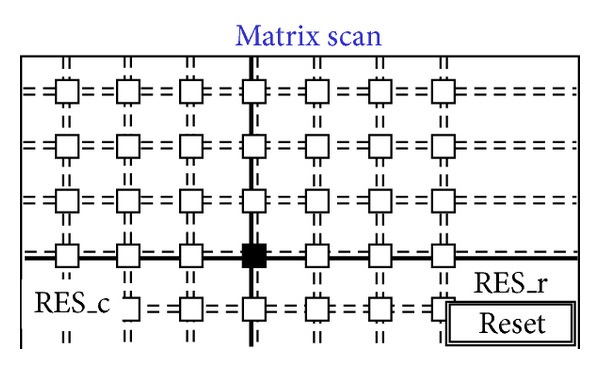
Last reset on the column.

We now provide a short example by following Figures 7 to 14.  Figure 8 shows a situation with five black pixels—that is, hits—where active wired-or conditions cause the activation of 3 OR_c wires. These three wires are bold lines in Figure 7. This corresponds to the sample phase of Table 1 (〈Pixel〉 is the pixel's output value).  Figure 8 shows that only the column furthest to the left (second left in the figure) containing at least one hit is enabled via the bold RES_c lines. In particular, the RES_c bold lines mask all the other columns except the one to be read out. This is the hold-mask phase of Table 1. In addition Figure 9 shows, via the OR_r bold lines, which pixels on the selected column have hits (black-colored pixels) and which pixels do not (white-colored). The gray-colored pixels are hits still to be read as their columns are masked out. They will be read out later. This is the hold-read phase of Table 1. This column can then be reset via RES_c and RES_r parallel assertion as shown by the bold lines of Figure 10. This is the reset phase of Table 1 and the black pixels are going to be reset. The process then moves one column to the right where another two hits are present. In fact Figure 11 shows the two black-colored pixels belonging to a new column (third left in the diagrams). These are the next hits to be read. If there had been blank columns in between, they would have been skipped by the readout control unit (not shown in the figures as it is not a significant point).  Figure 12 shows the reset phase of the new column after the hold-read shown in Figure 11. Then Figures 13 and 14 show the hold-read and reset phases for the last enabled column. This holds one hit (fourth left column in the figures).

Thus, the columns are read out one at a time and all hits belonging to the same column are read in parallel and independently of the number of the rows and columns not containing hits, which are ignored. In principle, the entire readout phase takes twice as many clock periods as the number of columns that have hits. It follows therefore that the two hold-read and reset phases are the only two cycles needed to enable and read out an entire column of pixels.

It should be noted that during all readout steps, the entire matrix must not be necessarily frozen to avoid event overlaps [63–66]. Only the active columns, those involved in the readout process, have to be frozen. Hence, specifically for the active columns, once a given event has produced hits, these must be read out before a new event produces its own hits otherwise all the hits would overlap and this does not allow for the reconstruction of individual offline events. To overcome this, as in normal practice, each event is associated with a time stamp to reconstruct the hits according to the time they were produced. The readout electronics create a time stamp via a digital counter which increments on a bunch of external signals. This counter is unique for the entire matrix. As the pixel logic does not require any internal register, time stamps can be saved outside the matrix of pixels within a less congested area.

A pixel detector is also characterized by its dead time. Here, the dead time refers to the time interval required by the sensor and its in-pixel amplification and readout logic to return to its full sensitive capabilities after having been hit, frozen, and, eventually, read out via the digital architecture that is located outside the matrix. It should be noted that one aspect is the inherent recovery time required by the in-pixel sensor-amplifier-shaper-latch circuit to return to its initial condition, while another is the time required by the external (off-matrix) readout logic to scan the matrix and reset a frozen pixel. This latter time can be quite long if the readout architecture is not very efficient and can determine the overall dead time of the sensors and, consequently, of the system. This is why we have investigated a circuit to reduce the overall dead time as close as possible to the pixel inherent value, whatever it is, by increasing the off-pixel efficiency of the readout architecture. Readout systems that use serial architectures (for charge-coupled devices or general applications [67–70]) are not acceptable because of their low speed; for some HEPE readout times of the order of 1 *μ*s are too high. Thus, here “fast” refers to readout times of the order of 1 *μ*s or less. The total readout time for a generic matrix will depend on several variables. The number of pixels, the average matrix occupancy, the master clock frequency, and the pixel switch-off time [71, 72] could be the main parameters for assessing the readout response of a pixel device [73, 74]. However, by considering the modern readout electronics applied in pixels detectors at LHC, based on token-passing techniques [75] and on offline digital sparsification circuits, the approach we propose could achieve good timing resolutions. Just to provide a general description, whatever the matrix of sensors, if the pixels are arranged on token-passing architecture, they are logically organized on a quasiserial topology with information being transmitted sequentially from one pixel to another. There is a control token circulating through the rows and columns of the matrix. It moves horizontally along the columns and, in the event of one column having hits, the token enters into the column and starts moving vertically along the rows. When the token has scanned all the rows it can start moving along the next columns again. Of course, depending on the specific application, the token-passing architecture can be enhanced. Empty pixels can be easily skipped. However, in the solution we describe here, as the matrix is only swept along the columns, it is as if the token was just horizontally passed through the columns and never vertically through the rows. In addition, any stop along the columns lasts only two clock periods. This explains why as many hits as the number of rows can be read out in parallel at a time, increasing the overall speed of the system. In addition, the larger the matrix, the more efficient the system. Of course larger matrices have other problems related to the distribution of the pixels over a wide area. For example, this leads to an inherent dispersion of the characteristics of the pixels due to silicon nonhomogeneity and voltage drop along long power lines.

For example, HEPE readout systems of pixel detectors read out matrices of thousands of pixels with an average occupancy of just a few percentage points (or lower, see below) leading to some tens of hits per bunch crossing. These hits are grouped mainly in clusters over the noise. If the proposed technique is used to read out these systems, the average readout time can decrease to several hundred ns—recent readout architectures implemented in HEPE and some proposed solution for future experiments, such as ILC, have readout times above 1 *μ*s [75, 76].

The proposed technique points to a promising readout speed compared to those recently obtained in HEPE. In more detail, when a pixel device is forced into resetting, even if external reset signal is immediate, it is possible that the time required to release the charge accumulated into its internal data acquisition chain (charge amplifier [77–80], shaper [81–83]) may take longer (by as many as several *μ*s). This is the time a pixel could require to return to its initial state after having been hit, read out, and reset. This leads to inevitable system inefficiency depending on hit rate, pixel area, and pixel reset time. However, modern pixel devices applied in HEPE reach inherent efficiencies over 99% (99.9% to 99.99%), handle hit rates of the order of tens of MHz/cm^2^, pixel areas of the order of 10^3^ ÷ 10^4^ *μ*m^2^, and reset time of the order of 1 *μ*s. Thus, a readout time of some hundreds of ns can also be seen as competitive in terms of system efficiency.

## 6. Typical Readout Efficiency

Nowadays, the vast majority of front-end circuits developed for silicon detectors focuses on energy measurement. The detected charge is distributed as a Landau [84, 85] distribution centered at 2.4 fC, corresponding to a minimum ionizing particle (MIP) [86] in a 200 *μ*m thick silicon detector. In other words, in particle physics, ionizing particles cross the solid-state (generally made of silicon) detectors, leaving a minimum amount of charge, by minimally interacting with the matter. On the other hand, this is one of the scopes of the detector, which must detect the particle by interacting as low as possible with it. In this way the particle trajectory is left almost unchanged. Hence, the average deposited charge, spread as a Landau distribution, is of the order of few thousands electrons, that is, a few fC. The deposited charge varies linearly as the length of the crossing path, and for a MIP this charge is estimated in about 80 electrons per *μ*m. Such a small charge, eventually, is the one that must ignite the sensor of a pixel—somehow a reverse-biased p-n junction—and stored as binary or digital information within a detector. For a good efficiency the system must be able to detect signals from 1 fC to 10 fC with a time resolution better than 200 ps root-mean-square (RMS).

There are many methodologies to investigate the front-end readout efficiency [87–90]. First, there is inherent and unavoidable sensor inefficiency due to its blind time. In more detail, when a sensor collects charge it basically stores it in a capacitor that must be depleted at the end of the process. This process needs a given amount of time to complete, which can be estimated in the order of 1 *μ*s. During this time the sensor is blind if a new event deposits additional charge. The probability of a multiple event on the same sensor within a short time depends on the experiment particle rate, today on the order of hundreds of MHz per cm^2^, and on the speed of the readout electronics that is also responsible for the sensor reset. Moreover, apart from the sensor efficiency, which might be defined as the probability to detect an event—crossing particle—among all the actual events, which is above 99%, there is also efficiency due to the readout electronics. It is specifically the latter efficiency that is here described. In particular, readout efficiency depends on two main conditions.

- The ionizing particles that traverse a frozen structure are lost. The structure can be the entire pixel along with the sensor or a group of pixels such as macropixels, regions or zones of pixels that are described below.
- The queuing system that follows the front-end might go in overflow. Below is described a barrel structure that is used as a buffer to temporarily store the data during the data taking.

First condition occurs when a pixel has detected a particle, holds its space-time information, but it has not yet been read out. In some terms the structure is frozen. In this case, any other information provided by the sensor is lost as the following readout electronics is not capable of loading and holding other hit information. Conversely, the second condition occurs when there are too many pixels to be read out within a short time, and this depends on the input particle rate that is described by the Landau shape. If within the same time slot, the overall system readout electronics is not capable or freeing the buffers where the hit information have been stored, these buffers overflow and start losing data. Naturally, the buffer depths are designed to stand the input data rate with its distribution fluctuations, but it is not possible to guarantee to never go overflow. What is more, the two conditions can occur concurrently and hence even more decrease the total efficiency of the system.

## 7. Example of Efficiency Estimation

Let us give an example to estimate the efficiency of a system composed of a matrix of pixels and readout electronics.

Let us say that we have a matrix of 100 by 100 pixels, that is, 10.000 (10^4^) pixels, measuring 100 by 100 *μ*m^2^ each. We have a total area of 100.000.000 *μ*m^2^, that is, 10^8^ *μ*m^2^ or 1 cm^2^ of sensitive area. Let us also say that, on average, we expect 100 M, that is, 10^8^, ionizing particle per cm^2^ per second through the detector. This is the input average data rate. Say also that these particles are randomly and uniformly distributed over the sensor, that is, every pixel has the same probability to be fired, over time. Thus, we expect 10^4^ hits per pixel per second on each pixel, which is 100 M hit per second over the sensor of 1 cm^2^, or 0.01 hit per *μ*s over each pixel. Hence, the hit rate per pixel is, on average, 10^4^ Hz (10^8^ particles over 10^4^ pixels per second) or 10^−2^ hits per *μ*s, which means that the probability to have two hits at the same time slot of 1 *μ*s over the same pixel is 10^−4^. Or again, we have on average 1 hit on a given pixel every 10^−4 ^s that is the reciprocal of 10^4^ Hz.

Moreover, let us say that the dead time of each pixel is still 1 *μ*s, that is, it takes 1 *μ*s to recover after being fired. This means that 1 *μ*s over 10^−4 ^s of the time, which is 10^−2^, or 1% of the time, the pixel is blind.

This is the individual pixel inefficiency or the pixel efficiency is 99% due to its dead time. As a consequence of that, the entire system cannot be more efficient than 99%, and the overall efficiency is expected slightly lower than that. As said, the two above conditions can, in general, further reduce this number. However, the inherent dead or blind time of a sensor is always present, so that the other digital forms of inefficiencies can, and must, be reduced until they became negligible.

In the next paragraph we describe how system efficiency is studied, investigated, and how the technical parameters of readout electronics might be determined.

## 8. Innovative Readout Efficiency

A simulator has been designed to generate random hits and to investigate how the readout parameters can affect the speed of matrix of pixels. The number of hits that occur on average, within a time unit and on an area unit, is called hit rate. Then, a control system efficiency, which effectively means the relationship between the hit recorded by the sensors and the hits generated, monitors the correctness of the output data. Generally speaking, there are false or missing hits due to different reasons:

- one single particle deposits a given charge that diffuses over several pixels,
- a given particle does not release a sufficient charge underneath the sensitive area of the pixel,
- a given pixel has been previously masked out or frozen.

Depending on how the matrix is swept, hence depending on the readout clock frequency and on the freezing frequency, the efficiency can be estimated once the pixel rate is known in advance. In this section, a simulation for a hit rate that ranges from 100 Hz/pixel to 3 kHz/pixel, a readout clock frequency fixed at 40 MHz, typical of LHC and Super-LHC, and a freezing time of the matrix of 5 *μ*s are shown. This means that, on average, every 5 *μ*s the matrix is swept and the hits are read out. This also follows recent studies and developments to speed up the readout logic for electronics in physics applications [90–92].

The 40 MHz also fix the data throughput as it is here considered that one hit per clock period can exit the readout circuit. This is to avoid having parallel output ports that might lead the IO pads to expand too much.

**Figure 15:**
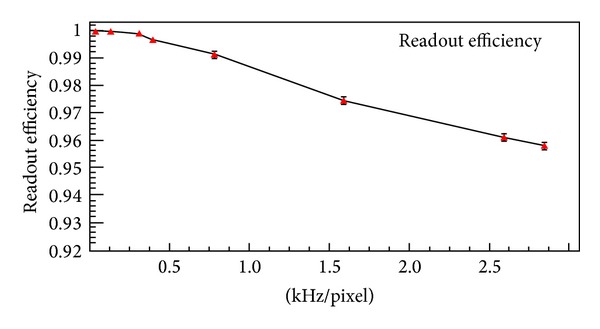
Efficiency versus hit rate (clk@40 MHz, Freezing-Time@5 *μ*s).

**Figure 16:**
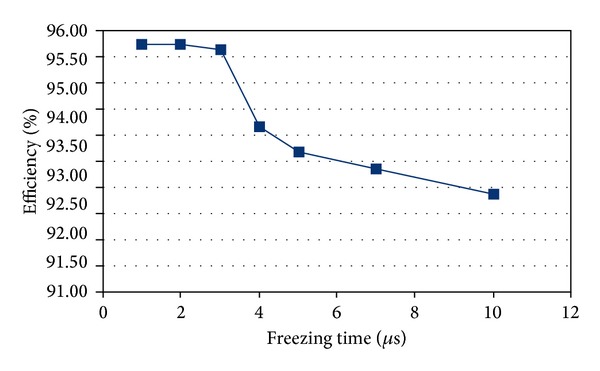
Efficiency versus sweeping time (clk@40 MHz, Hit-Rate@2.5 kHz/pixel).

In this context the area of the matrix and the dimensions of the pixels are not relevant provided the readout logic is able to handle the data throughput. So, everything is referred to the hit rate. Again, as an example, the simulator provides randomly distributed hits over the pixels with the expected hit rate.  Figure 15 shows how the efficiency drops depending on these parameters. It is visible that the proposed parallel readout maintains a reasonable efficiency for hit rates up to some hundreds of Hz per pixel. To overcome this limit a higher system readout clock is necessary. Another simulation has been carried out by sweeping the freezing time of the matrix while not changing the other specifications as above.  Figure 16 shows that it is here considered a low-occupancy of the matrix, whatever its dimension, so that the readout time of the matrix can be smaller than the freezing time. It is visible a knee showing how the smaller the freezing time, the higher the efficiency.

Hence, depending on the application and the constraints, the system can be tuned accordingly. The proposed solution from the architectural viewpoint uses first-in-first-out (FIFO) memories to store the data temporarily. Additionally, the FIFOs here require to be written via a variable length word depending on the number of hits present at the same time on the same column. This variable-length FIFO logic can be referred to as a circular barrel of storing elements. Particularly, a barrel depth from 16 to 32 leads to efficiency over 95%.

## 9. Organization of a Matrix of Pixels in Smaller Areas and Zones [93, 94]

**Figure 17:**
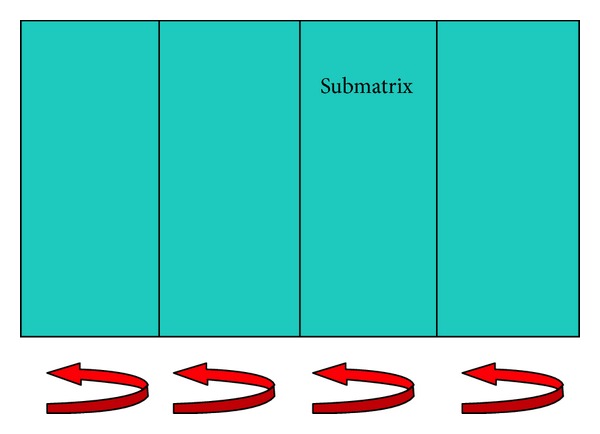
Matrix areas.

What have been described until now can be adapted not only to an entire matrix of pixels but also to some portions, which work in parallel, of a huge matrix. In this way an estimation of the system efficiency can be done separately for the portions of the matrix and, eventually, entirely on the full matrix.  Figure 17 shows how a large matrix of pixel can be divided into smaller matrices, still having the same height.

**Figure 18:**
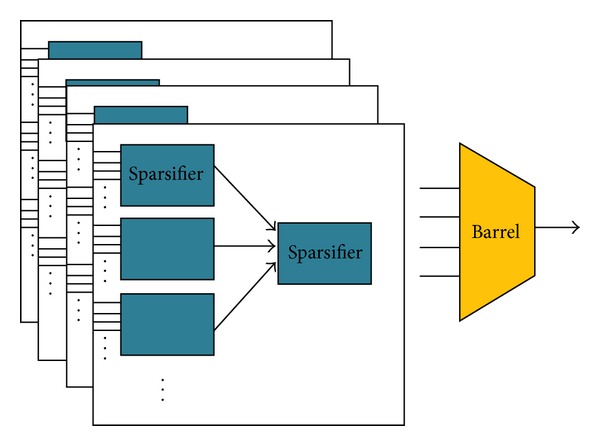
Sparsifiers and barrels.

The sparsification logic, and more precisely the sparsifier circuits, executes the parallel readout of the hits belonging to the same column of pixels. In this way all the submatrices may be swept concurrently since each one has its sparsifier circuits to read the hits in parallel as described above.  Figure 18 shows the blocks that read out, in this example, 64 pixels at a time. In addition, the 64 pixels that belong to a given column are grouped vertically into 8 zones of 8 pixels each. Hence, a sparsifier works on 8 zones in parallel, and each hit generated refers to a zone, including the zone address and the zone hit pattern. This technique was thought foreseeing the generation of clusters for any impinging particle.

As mentioned, any sweep of the matrix is associated with a given time information. This latter can be either provided via a classic triggering system or through a cyclic predefined logic as for a data-driven trigger. The proposed readout architecture can work with both triggering systems. Hence, the hits are swept and queued depending on their time information, without mixing data belonging to different events. In particular, the hit information, after being formatted with space and time coordinates, enters a queuing system. A particular queuing system has been developed since several hits at a time need to be stored. A variable input-width barrel system is the best candidate for this scope. It works like an asymmetric FIFO where the input port width is a multiple of the output port width, in addition this multiplicity is variable and it is determined at each write operation by the sparsifier that knows how many hits need to be stored. This component basically implements the required buffer in which a variable number of hits can be written contemporaneously.

**Figure 19:**
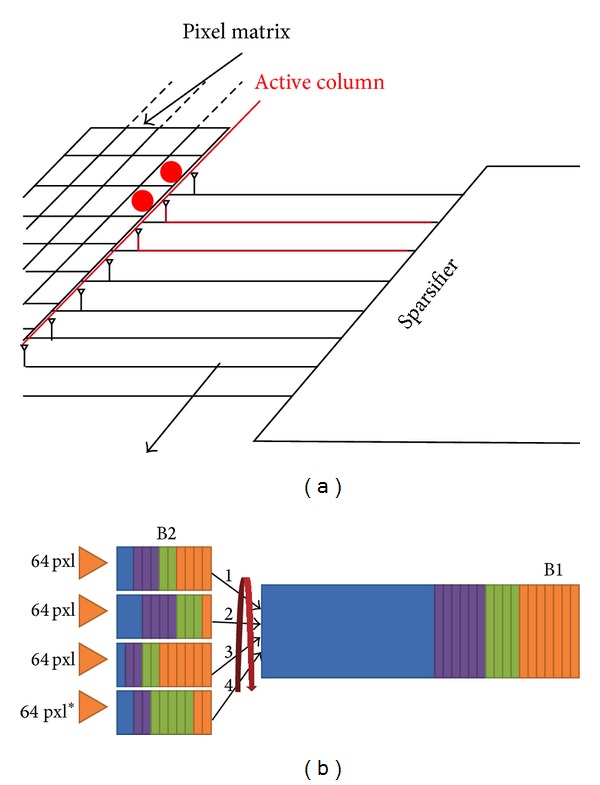
Sparsified data and barrel filling.

Hence, provided the barrel system has a sufficient depth, the hits data can be stored immediately when they are read out via the sparsifiers, independently of their cardinality. Figures 17 and 18 show a configuration with 4 barrels—B2 in Figure 19—that can receive hits from portions of matrices with a depth of 64 rows each. The barrels show different event data by a different color scale, each event is associated with specific time information corresponding to a matrix sweep. It is evident that each event is stored into a compact portion of each barrel. Then, the four B2 barrels convey the data to a further deeper barrel—B1 in Figure 19—while still maintaining the time sorting of the events. Top of Figure 19 also shows how the sparsifiers work on the active column. The active column, instead, is a concept concerning the readout of the hits.

It can be said that a column of pixels is divided into several vertical zones and each sparsifier works in parallel on 8 zones. Let us define *Z* the zone dimension, Bd the barrels' depth, Nb the number of barrels (= number of sparsifiers), and *H* the number of bits that compose a hit. In this way the total number *M* of bits used to store the hits into a FIFO queuing system is simply given by the product

(1) $$\begin{array}{c} M=\text{Nb}*\text{Bd}*H. \end{array}$$

The number of required barrels

(2) $$\begin{array}{c} \text{Nb}=\frac{\text{number}\;\text{of}\;\text{rows}}{\left(8*Z\right)}, \end{array}$$

in fact each barrel receives hits from 8 adjacent zones, each made up of *Z* pixels. The length of a hit *H* can be expressed like

(3) $$\begin{array}{c} H=K-\log_{2}\left(Z\right)+Z, \end{array}$$

where *K* is the length of a classic *X*-*Y* full-resolution sparsified hit. The zone-sparsified hit is Log_2_(*Z*) bit shorter since the zone address has a bigger granularity than the pixel address, but at the same time the hit is *Z* bit longer since it includes the zone hit pattern. For what concerns the barrel depth Bd, generally speaking it holds that

(4) $$\begin{array}{c} \text{Bd}=\text{Bd}\left(\text{hit rate}\left(Z\right)\right), \end{array}$$

but in normal working conditions, when the mean input rate is far smaller than the barrel throughput, it can be shown that the input hit rate does not depend on the zone size *Z*. It follows that neither Bd depends on it. Once *M* is expressed in function of *Z*, it is easy to see that the zone technique brings a significant reduction in the required on-chip buffer memory, reducing the overall readout silicon area.

**Figure 20:**
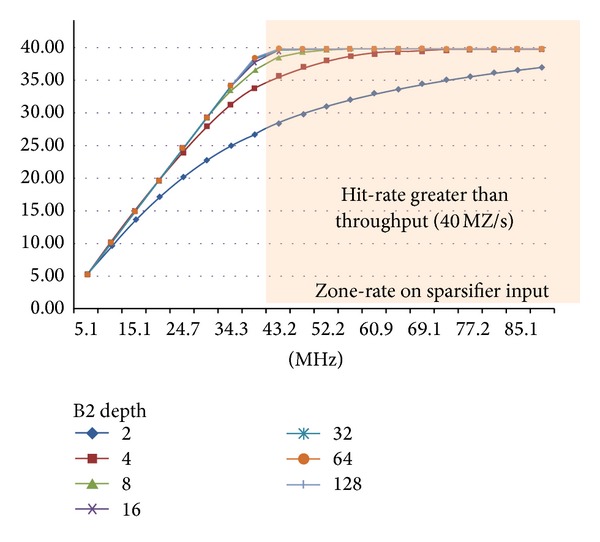
Barrel depth simulations: input versus output rates.

The evaluation of an optimal barrel depth is a delicate point since it deals with the distribution in time of the hit and not only with the mean hit rate.  Figure 20 refers to mega-zones per second, MZ/s and to the B2 barrel depths. To optimize also the barrel depth, a simulation has been carried out and Figure 20 shows how the barrel depth affects the system efficiency. It is shown that when the mean input-rate overcomes 40 MHz the output-rate—throughput—can no longer empty the barrels and, consequently, the barrel bandwidth is saturated. In this case the barrel efficiency, estimated as the ratio between the output and the input rate, starts to decrease like 1/*x* as the input-rate increase. However, on the left hand side of the figure, where the mean rate is smaller than the barrel throughput, the curves only approximate to a straight line. Better approximation is reached with deeper barrels, which can buffer more hits during rate fluctuations over the average.

## 10. Example of a 320 × 256 Pixel Matrix

**Figure 21:**
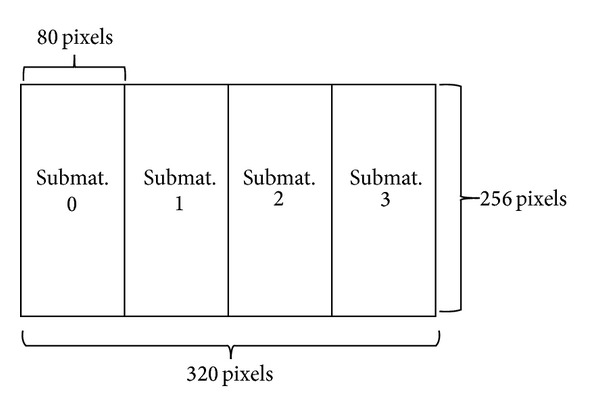
Matrix partitions.

The matrix considered for our readout architecture is 320 × 256 pixel wide, for a total of about 81 kpixels.  Figure 21 shows how this sensor array is divided into 4 smaller matrices (80 × 256), each one served by a dedicated and independent readout. With a 40 *μ*m pitch, the total sensor area is about 1.3 cm^2^, but it can reach 2 cm^2^ using 50 *μ*m pitch.

The major problem with high-density matrices is the interconnection of the readout block with pixels. In general, the matrix is squared (or rectangular) and the input-output contacts are placed at the bottom of the matrix, and at one side only. Consequently, if we double the number of contacts (bondings) of that side, the area available for the pixels can increase to the fourth power. The consequent upper bound in the matrix dimension is the limited interconnection density in the contact side of the two blocks. In order to decrease the number of interconnections between sensor and readout, and hence to increase the matrix dimension, we introduced the concept of macropixel (MP).

**Figure 22:**
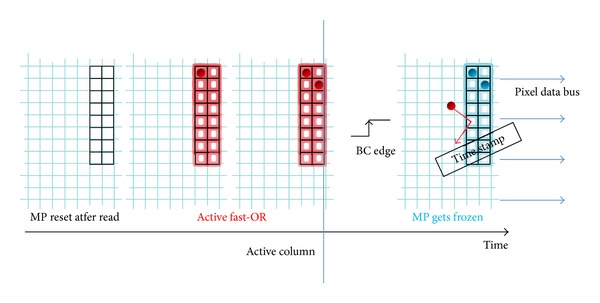
MP working phases.

The MP is an independent group of pixels, with a local fast-or line, that is, a logic OR of all the pixel outputs, connected to the readout logic. If the fast-or line is activated, it means that at least one of the pixels inside the MP has been hit. Here a MP dimension of 2 by 8 is taken and hence the entire matrix is composed of up of 5120 MPs. On the arrival of a bunch crossing clock (BC) rising edge, the content of a fired MP is immediately frozen by the logic, and no more pixels can be turned on within the MP, even if a signal over threshold is detected (see Figure 22). BC clock beats time in the experiment and it specifies the time granularity of the events recorded. For this reason a modulo 256 counter has been implemented in the readout logic, incrementing on each BC positive edge. When a MP is frozen, it is associated with the current value of the time counter. Thereafter it waits to be read, reset, and reactivated. Timing information is recorded by the readout logic when a MP is frozen.

The hits are read through a column-wide common bus, called pixel data bus, shared among all the pixel columns. Thus, the active column of pixels, which is intended to drive the pixel data bus, is selected, see Figure 22, by imaging that the active column moves from left to right along the frozen MPs.

**Figure 23:**
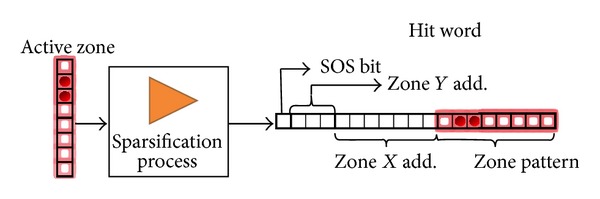
Zone sparsification diagram.

Figure 23 shows how the content of a MP is read out, by selecting the correct MP row column-pair. The current active column drives the pixel data bus that is analyzed by the sparsifiers. Their task is to encode the space coordinates of the hits into hit words. Sparsified data is then stored in a formatted asymmetric FIFO such as the barrel. The sparsifiers encode also the information about the beginning of a matrix scan. When a new scan starts, each sparsifier stores a special word containing the associated time stamp in its adjacent barrel. In the considered submatrix, we have 256 rows of pixels and thus a 256 bit wide pixel data bus. The sparsifier has a 64 bit wide input bus, and it is able to process the entire column in one clock cycle. In the proposed architecture 4 sparsifiers working in parallel are implemented to cover the full pixel data bus. To profit from possible clustering of hits, the sparsification is not done at the pixel level. The 64 bit sparsifier input bus is divided into 8 bit segment zones. A hit pixel in a certain zone generates hit words containing information of the entire zone. A hit word consists of the *X*-*Y* zone addresses plus the zone hit pattern. In case two MPs, belonging to different time stamps, are fired on the same columns, it is possible to read only the desired one leaving the other waiting for next sweep. This allows, for example, to read out only those MPs tagged with a given time stamp, permitting a timewise sweep of the matrix.

## 11. Simulation Results

**Figure 24:**
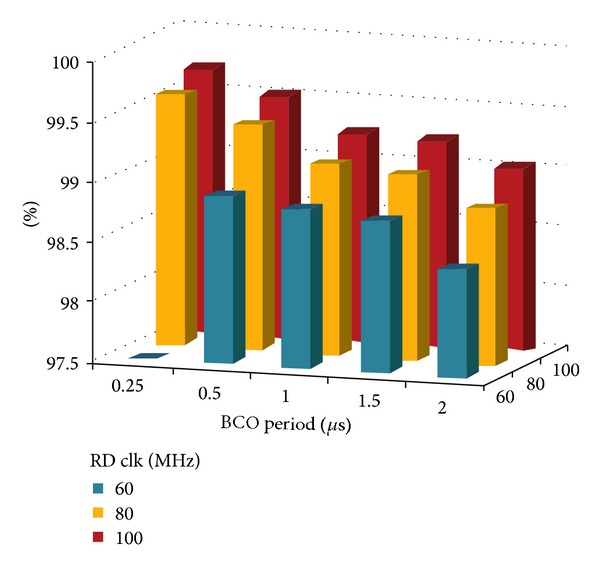
Freezing efficiency plot. The efficiency drop in lower-left corner is due to scan buffer overflows. This implies no hit loss but a longer average sweeping time and a reduced time resolution for some events. Freezing efficiency results in %. 1 *μ*s simulated at 100 MHz/cm^2^, corresponding to more than 30 khit generated on a 80 × 256 submatrix, 40 *μ*m pitch, no clustering.

The architecture shown has been implemented with a synthesizable VHDL model. Test bench simulations have been carried out for model verification and fine adjustments of parameters. An intensive simulation campaign was performed also in order to establish the efficiencies of the readout architecture. First of all a test bench was set up for the evaluation of efficiencies concerning individual submatrix readout. A nonsynthesizable MP VHDL model was realized with random hit generation capability, adjustable rate and shape, and provided with built-in efficiency trackers. The submatrix model is a 2D array of MPs with parameterized dimensions. A span of typical working conditions has been probed, ranging on realistic clock frequency intervals and hit rates. The results presented in Table 2 and plotted in Figure 24 refer to a set of simulations carried out with a constant hit rate fixed to the target value of 100 MHz/cm^2^. The longer is the average freezing time, the lower the efficiency. These values do not take into account the possible inefficiencies of the sensor and it is supposed that each MP is ready to trigger right after the reset. Freezing inefficiency is then a factor of the total inefficiency caused directly and only by the readout algorithm and the matrix architecture. It represents then a good benchmark of how well the architecture is behaving regardless of all the other sources of inefficiency. We varied the main read clock—RDclk in Figure 24—of the digital readout from a minimal value of 60 MHz to a max value of 100 MHz, with a middle step of 80 MHz At the same time we varied the BC clock period (time granularity) from 0.25 *μ*s to 2 *μ*s.

**Figure 25:**
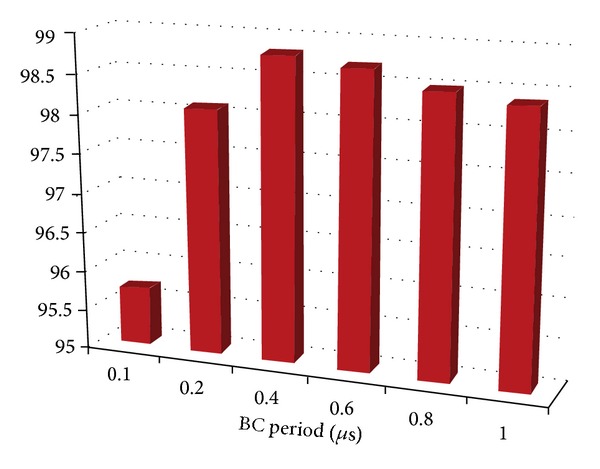
Freezing efficiency plot for the whole matrix. Efficiency drop at 100 ns is caused by scan buffer overflows. On vertical axis we have efficiency expressed in %.

A second campaign of simulations was intended to test the behavior of the entire chip, putting together 4 submatrices and 4 readout instances plus the data concentrator. The full 82 Kpixel matrix and the 4 independent instances of readout were simulated at a real time rendering factor of about 150 ns per second. For this simulations we imposed the usual hit rate of 100 MHz/cm^2^ and we used a 66.6 MHz read clock and a 200 MHz fast clock for the output bus driving. At the same time we wanted to inspect the behavior of the whole infrastructure scaling the BC period down to hundreds of ns. Results are reported in Figure 25.

## 12. Fast Readouts in ASIC Prototypes for Scientific Applications

In the late 1980s and early 1990s, when the US Superconducting Super Collider (SSC) and CERN Large Hadron Collider (LHC) R&D programs were started it was already known that the successful completion of experiments would have required new Application Specific Integrated Circuits (ASICs) to provide fast detector readout and ondetector, online data compression. In addition, radiation hardened components were required to stand the harsh radiation environment of the high-energy physics (HEP) detectors.

In the 1990s specifications for military and space environments already existed, and a number of vendors had certified production lines (in 1997, 20 vendors had production lines certified according to US Department of Defense MIL-PRF-38585 specification [95]). However, as semiconductor industry was never driven by HEP applications, these components had to be designed and fabricated by the HEP community. Eventually, the technology of choice for the LHC ASICs up to the year 1999 was the DMILL 0.8 *μ*m BiCMOS process [96] even if some of the chips were also produced in other technologies [97]. In the meantime, the requirements of microprocessor and telecommunication applications pushed the semiconductor industry into a new age of deep submicron technologies. Due to small MOS gate oxide thickness (typical *≈* 5 nm for 0.25 *μ*m technology) the deep submicron technologies are to some extent naturally resistant to radiation (tunneling electrons remove the positive charge accumulated in the gate oxide). The implementation of design elements to eliminate radiation induced leakage currents (guard rings, enclosed layout transistors—ELTs) permitted the MOS devices to reach a very high level of radiation hardness [98, 99]. Typical ASICs examples are DTMROC [100, 101] and APV [102, 103]. Starting from year 2000 the technology of choice for HEP applications was 0.25 *μ*m CMOS [104–107]. Note that dedicated standard cell libraries were developed for both technologies (BiCMOS 0.8 *μ*m DMILL and 0.25 *μ*m CMOS) [98, 100].

In fact, the position of the ASICs in the closest proximity of the detectors introduces certain requirements for the designers, namely, the following.

- The ASICs should have low power consumption. The mass of cooling systems necessary to provide heat removal depends on its cooling power, and the mass of powering cables depends on current consumption. Generally speaking, the mass of the system should be minimized as it distorts particle tracks and makes the system resolution lower. For the same reason, shielding the ASICs against radiation is not applicable.
- The ASICs should be of minimum size and/or size adjusted to the detectors. The size of the ASICs limits the size of the supporting boards, which should also be minimized for the reason described above.
- The data processing ASICs should provide many readout channels to minimize the size of the whole system.
- ASICs that process the data from the sensors should provide data compression. The first level of compression is usually a selection of the data based on its potential physical importance. A dedicated system (trigger system) is used to estimate in real time, if there is an interesting event in a given detector sector. All the front-end ASICs have to store the data waiting for the decision of the trigger system. Then the stored data, after being confirmed by the trigger signal, are sent out for further analysis. Otherwise they are discarded.
- The ASICs have to be radiation hard. The problem of radiation hardness of the ASICs in HEP can be treated as another problem of reliability. The main difference is the size of the system: in HEP experiments thousands of ASICs have to work synchronously to provide proper particle identification. Any desynchronization or malfunction (for example due to a SEU) leads to data loss. The number of lost data depends on a number of parameters and is very difficult to estimate at the design level. The complete system reliability can be verified only after full system tests. However, at the design level there are a number of possibilities for enhancing system reliability by improving the ASICs radiation hardness. As mentioned before, for deep submicron CMOS technologies the single event effects are the main issue. Using guard-rings and ELTs is enough to gain TID radiation hardness for the most demanding HEP applications.

Below, a prototype ASIC [108] is presented as it has been designed with the intention to match the future HEP requirements, with high input data rate and fast readout approaches. In addition, the chip was designed also to fit data-driven [109, 110] experiments, besides the more common triggered systems. The difference relies on the decision between useful data and hits to be rejected. In general, triggered systems have local buffers on the front-end electronics to save a few *μ*s of taken data, waiting for the delayed decision of the triggering system, which has a more complete view of the data coming from a larger part of the detector. Triggered systems handle a smaller amount of data, since part of these are rejected. The drawback is that some good data might be discharged by accident. By contrast, data-driven systems store all the incoming data from the detector but, again, they need very fast readout logic.

## 13. APSEL4D Chip: A 4096-Pixel Matrix with a Fast Data-Driven Readout Sparsification Circuit 

The circuit is a digital architecture for a sparsified readout that interfaces with a matrix of 4096 monolithic active pixel sensor (MAPS). It is the base for a prototype of a mixed-mode ASIC, namely, Apsel4D. It reads out and sparsifies the hits of a matrix of 4096 pixels. Once read, the hits are switched off. The matrix is divided into regions of 4 × 4 single pixels thus, 4096 pixels are clustered into 256 groups of 16 pixels each, herein-after named macropixels (MPs) [111, 112]. In addition, the matrix is arranged in 128 columns by 32 rows of single pixels or, from a different viewpoint, in 32 columns of MPs, called MCs, by 8 rows of MPs, called MRs. Basically, let us say that when the matrix has some hits (pixels that detect an over-threshold charge), it is swept from left to right and, at each clock period, all the hits present in a column of pixels, from 1 to 32, can be read out in parallel. This operation starts as long as a hardwired readout queue has free locations to temporarily store the information of the hits. In fact, the hits spatiat coordinates are associated also with a time information mark (time stamp [113–115]) and the overall formatted data are either sent to the output port, or temporarily stored in a FIFO-like memory in case the output port is busy. Thus, in principle, the architecture can read out the matrix up to 32 hits at a time in case they belong to the same column and can send the formatted data to the output but, at the same time, the output port can only accept one-hit information at a time, and this is why a queuing system is necessary.

Moreover, the global architecture might be considered as a circuit that can run in two different operating modes, called custom mode and digital mode. In fact, it can be connected to an actual full-custom matrix of MAPS or to a digital matrix emulator composed of standard cells. In the first case the pixels may only be switched on via striking particles while in the second case the digital matrix must be loaded during an initial slow-control phase. The two different implementations share the same matrix's I/O pins but can be selected and activated only one at a time. For both modes, before running, a slow-control phase is required to load an internal configuration. In particular, 256 mask signals should be provided to select the MPs, which are to be read and which are not, for example, in case they are too noisy or broken (MP-mask). Other 32 masks can deactivate any row of pixels (ROW-mask). Default mask, after a reset phase, is all-at-1, meaning no-mask both for MP-masks and for ROW-masks. Any ROW-mask is to avoid that a single burned pixel invalidates the entire MR it belongs to. Moreover, it must be selected which of the two operating modes is wanted and, consequently, which matrix is to be enabled. The default mode, after a reset phase, is the digital mode. In addition, only for the digitally emulated matrix, 256 registers should be loaded to simulate a given charge injection over the silicon area. Default registers, after a reset phase, are all-at-0, meaning no hits. The readout circuit operates in the same manner for the two modes. Figure 24 shows a sketch of the two operating modes of Apsel4D.

This is valid for both custom mode and digital mode. The entire matrix of Figure 23, composed of 4096 pixels, is to be interpreted as follows:

- 32 MCs, addressed from left to right, range from 31 to 0,
- 32 rows of pixels, addressed from top to bottom, range from 31 to 0,
- 128 columns of pixels, addressed from left to right, range from 127 to 0,
- 4 columns of pixels inside each MP, from left to right, range from 3 to 0.

In this view each pixel is identified with a given MC, a given column inside the MC, and a pixel row. By converting these coordinated in digital logic it turns out 5 + 2 + 5 bits, that is, 12 bits altogether which address exactly 4096 pixels. This is the way the addresses are sent to the readout output port.

The entire readout circuit is divided into the following logical blocks of Figure 28. All the circuits are described along this document but the custom matrix. This latter is logically equivalent to the dummy matrix and, for the readout viewpoint, behaves in the same way.

By looking at Figures 27 and 28, the matrix (dummy or custom) is swept as follows:

- the matrix is always swept from left to right and only the frozen MPs are considered,
- the 4 sparsifiers work in parallel and cope with 8 rows of pixels each, out of the 32 rows,
- at any time one only column of pixels is considered; thus, it is not possible that different sparsifiers read different columns at the same time,
- at any clock time, at most, 8 hits per sparsifier can be read, leading to 32 hits altogether. Nevertheless, if this is the case, the successive barrels force very soon the standby condition as if a whole column is lit, this is a consequence of very high (local) occupancies which are noncompatible with the output port that outputs one hit only at a time. The event of local high occupancy can be handled as long as the average hit rate is smaller than the throughput,
- the sparsifiers have a depth of 32. However, considering that up to 8 hit per sparsifier might be loaded every clock cycle, the standby condition must be forced in advance even though the queues are not yet completely full,
- the standby condition can be forced both by the 4 barrels or and by the barrel-final.

The entire circuit, whenever it is not able to store the data provided by the sparsifier or sparsifier-out circuits, stops the sweeping of the pixel columns. This happens when the number of free location in the 4 barrel or barrel-final circuits is smaller than the number of data that are going to be stored. In such a situation the readout stops for 32 (barrel/barrel-final depth) clock periods until at least the queuing system of the barrel-final circuits is empty for sure.

In this design the standby condition can occur if at least one of the following two different events occurs.

(1) The average hit rate of the whole matrix (lit pixels over unit time) is slightly higher that the throughput—1 hit per clock cycle—and only the barrel-final is overloaded. This means that each single barrel can afford the rate over 1/4 of the matrix and the following inequality is satisfied:

(5) 14∗average-hit  rate  <throughput  (i.e.  1  hit  per  clock  cycle⁡)  <average-hit  rate.

In this case is only the barrel-final that forces the standby condition.

(2) The average hit rate is sufficiently higher than the throughput (1 hit per clock cycle) so that any of the barrels are overloaded. This means that neither of the barrels can afford the rate over 1/4 of the matrix and the following inequality is satisfied:

(6) 14∗average-hit  rate  >throughput  (i.e.  1  hit  per  clock  cycle⁡).

This case is only the first barrel that reaches the full condition and forces a standby signal.

However, for the readout viewpoint, no matter who interrupted the scan of the matrix via a standby request. The scan is halted for 32 clock periods.

The MC-address-decoder provides the address of the column of pixels, while the matrix readout is ongoing. It stops only over the MCs that have at least one hit. The readout of a MC lasts 5 clock periods, 4 to read out the columns inside the MC and 1 to reset the MPs just read. It can last more periods if the readout enters a standby condition.

**Figure 26:**
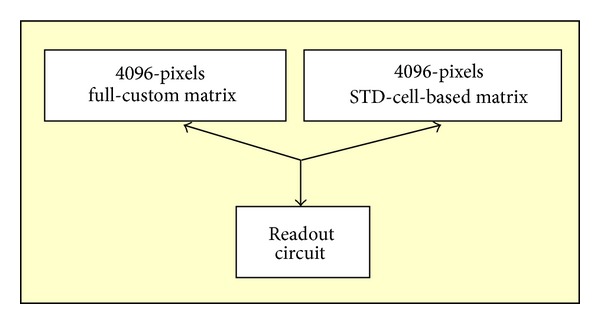
Apsel4D operating modes: custom mode and digital mode.

**Figure 27:**
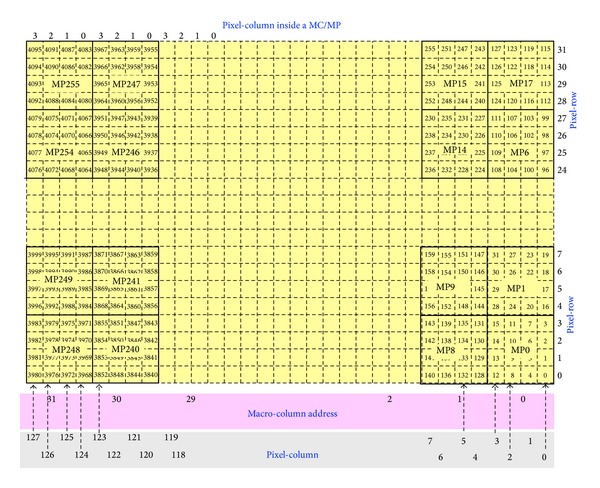
4096-pixel matrix.

**Figure 28:**
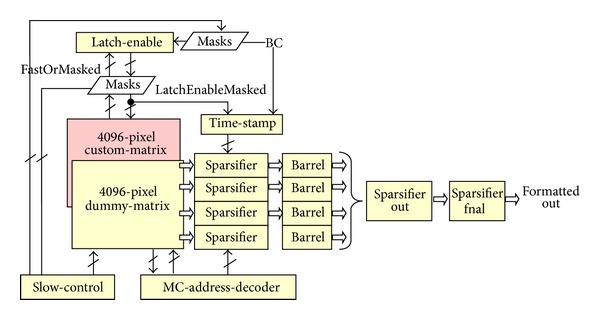
Readout architecture.

The barrel circuit provides a queue for the output data. As the entire architecture reads out at most one valid 21 bit word at a time, that is, at a clock cycle, in case more than one hit is read in parallel from the matrix, the exceeding hits must be temporarily held into a FIFO-like memory. This memory is a barrel that can be written with 1 to 8 24 bit words and can be read one location at a time. The barrel depth is 32: all in all it has 32 locations of 24 bit words even though just a subset of the overall bits is used. Each barrel reads only the data that originated from two MRs and, eventually, 4 barrels are designed to work in parallel to read out up to 32 pixel rows at a time as shown in Figure 26.

The barrel final circuit provides a queue for the output data that come from the 4 parallel barrels through the 4 sparsifier and the sparsifier-out circuits. As this architecture reads out at most one valid 21 bit word at a time, that is, at a clock cycle, in case more than one hit is read in parallel from the matrix, the exceeding hits must be temporarily held into a FIFO-like memory. This memory is a barrel that can be written with 1 to 4 24 bit words (4 Barrels) and can be read one location at a time. The barrel depth is 32: all in all it has 32 locations of 24 bit words even though just a subset of the overall bits is used.

**Figure 29:**
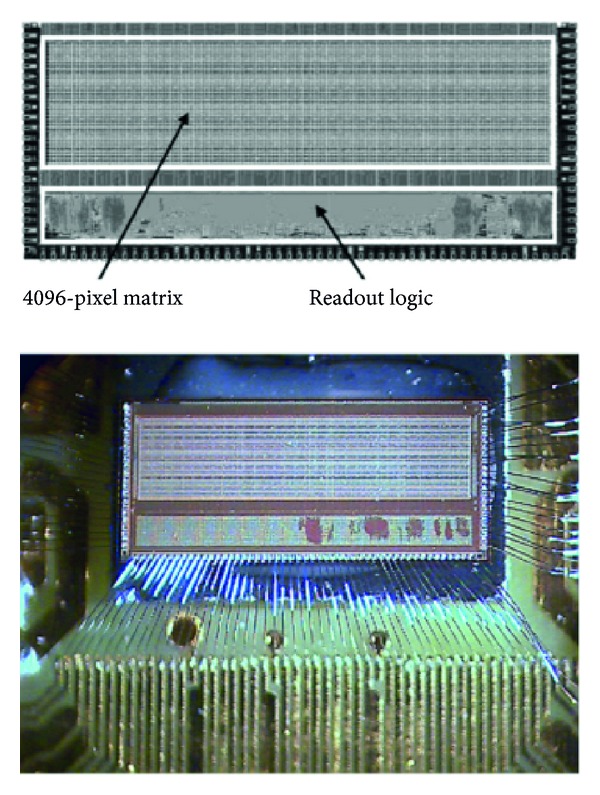
Apsel4D photo.

Figure 29 shows a microphotograph of the fabricated chip with emphasis to the matrix and readout areas.

## 14. Application of Apsel4D with Emphasis on the Readout Speed

The single electron two-slit interference is one of the most effective experiments to investigate the wave behavior of material particles [116–118]. The superposition of electron waves has been demonstrated many times in the past: single slit, single hole, double slit, double hole, multiple slits, and electrostatic biprism. Here, after having calibrated and tuned a Philips EM400T transmission electron microscope (TEM) via preliminary diffraction patterns of a thin wire and a carbon grating, we build up the time distribution of high statistics single electron interference pattern exploiting the Young-Feynman set up. For this, two nanoslits were prepared via modern nanotechnology tools and the TEM is used as a versatile optical bench. In addition, a recording system sensitive to single electrons replaces the final sensitive film of the microscope. In fact, our detector is based on a custom CMOS monolithic active pixel sensors (MAPS) composed of 4096 pixels, designed within the SLIM5 Italian [119–121] collaboration for application in vertex detectors of future high-energy physics colliders. The detector is equipped with a fast readout circuit able to manage up to 106 frames, of 4096 pixels each—even if most of them are empty—, per second (fps). We were able to collect high statistics samples of single-electron events while maintaining stable operation and the coherence conditions of the microscope. All in all, the large fraction of empty events allowed us to accurately measure the time distribution of electron arrivals.

This additional measurement of the time distribution was not generally carried out in standard electron imaging via pixel devices [122, 123]. At any time the matrix may have hits, along with their time stamps, belonging to different “events” and the readout process continues till all the hits have been read out.

The chip has been studied extensively on proton and pion beam tests at CERN [119, 120], where spatial resolutions compatible with 50/*√*12 *μ*m have been observed [124, 125].

Runs performed with 2.5 MHz of BCO clock frequencies (400 ns period) have demonstrated the capability of the chip to stand continuously 10^6^ frame-per-second to stand continuously the 106 frame-per-second (fps), although, in the experimental conditions of the present data taking, a much more conservative BCO frequency of 6.25 kHz has been used (160 *μ*s period).

**Figure 30:**
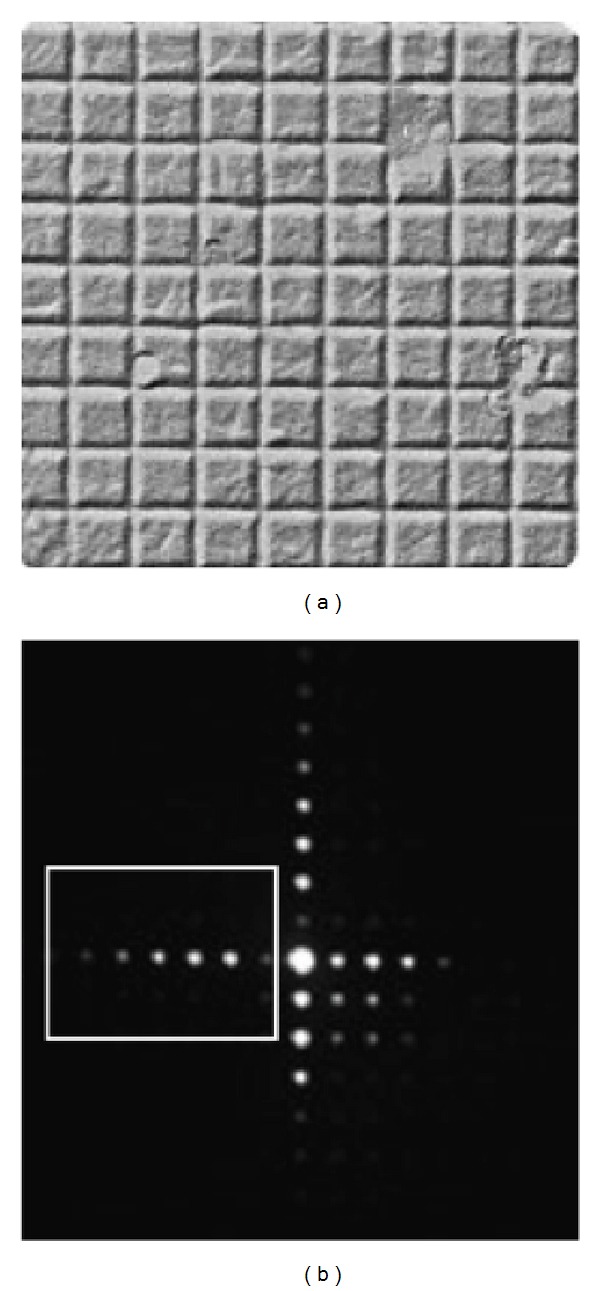
Typical diffraction grating image and a low-angle diffraction pattern: the white box enhances the portion of the pattern measured via the sensor.

**Figure 31:**
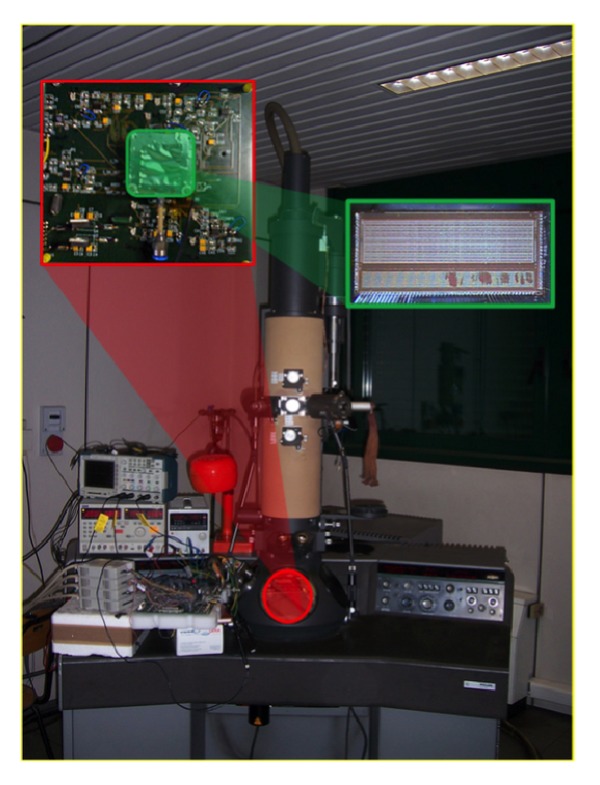
The transmission electron microscope (TEM), board and chip used in the experiment.

**Figure 32:**
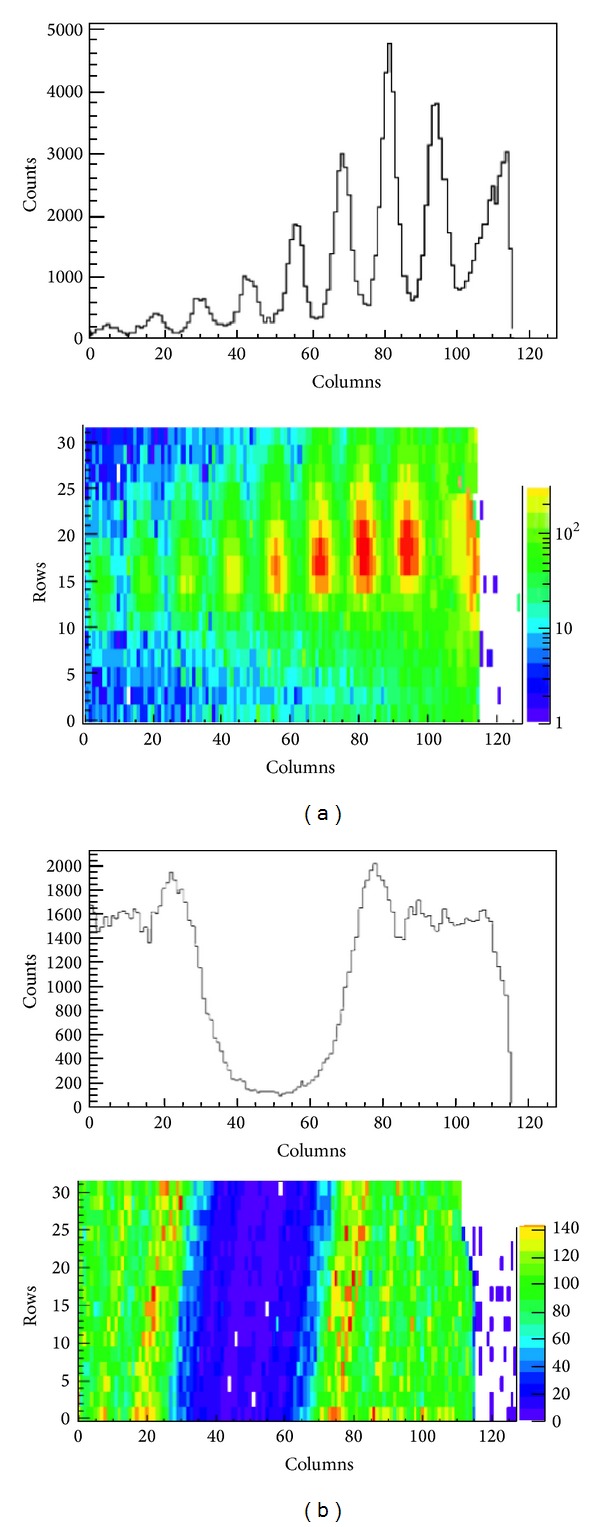
(a) Carbon grating image. Fringes on top. (b) Wire image. Fringes on top.

As we wanted to use a TEM to collect interference and diffraction patterns via a small matrix of pixels, we first wanted to calibrate the system and the data acquisition chain. For this aim we first used, as specimen, a carbon grating with a spacing of 463 nm, that is, very similar to the slit spacing described below.  Figure 30 shows a conventional image and a low-angle grating diffraction pattern whilst the entire data acquisition system, along with the TEM, is shown in Figure 31. The photograph of the sensor is zoomed out. In this experiment the average number of electrons per frame was about 8, meaning that it was sufficient to increase the coherence and diminish the beam intensity to obtain the condition of a single electron per frame. In particular, the right side of Figure 30 shows a rectangular white box superimposed on the grating diffraction picture. The white box represents the portion of the (diffraction) pattern visible by our detection system. In fact, Figure 32(a) shows one branch of fringes of the diffraction pattern from a carbon grating, using our rectangular matrix of pixels. The single electron condition was also reproduced by inserting a thin wire—0.5 *μ*m diameter—as a specimen.  Figure 32(b) shows the image of the wire along with the hit distribution integrated in time. The picture shows the first lateral fringes of the diffraction pattern. Hence, through these first measurements we were able to calibrate and tune the TEM to obtain diffraction patterns on the small pixel sensor, which measures only 6.4 × 1.6 mm^2^.

**Figure 33:**
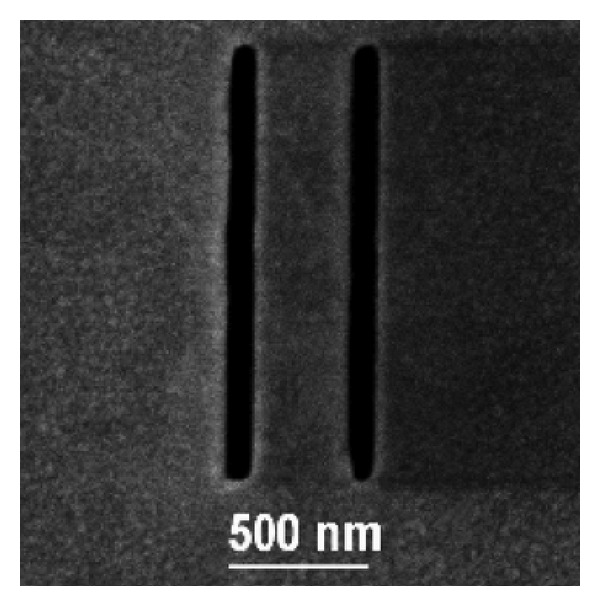
The nanometric slits.

These experiments were carried out with the Philips EM400T TEM, equipped with a hair-pin filament source operating mostly at 60 keV. These conditions lead to an equivalent De Broglie wavelength *λ* = 4.9 pm. The 60 keV value was a balance between the minimum detectable energy, otherwise we had no hits, and the features of the TEM. By considering this small wavelength value, the experiment required a dedicated set up. Hence, exploiting the electron lenses, it has been possible on one hand to demagnify the electron source so that coherence conditions were satisfied and, on the other hand, to magnify the image in such a way that its dimensions were compatible with the detector pixel size and numbers. In addition, due to the small electron diffraction angles (of the order of 10−5 rad) associated with the separation of the slits, the Fraunhofer pattern was observed in the so-called low-angle diffraction mode. In this electrooptical set up the condenser lenses were excited at their maximum strength in order to reach the necessary lateral coherence of the illumination and the objective lens is weakly excited in order to project the Fraunhofer image onto the selected area aperture plane. As a consequence, the microscope works as a diffraction camera having camera lengths extending up to several hundred meters. Then, the slits, shown in Figure 33, for the two-slits experiment were fabricated by a focused ion beam (FIB) milling a gold layer about 250 nm thick, deposited by flash evaporation on a commercial copper grid coated with a carbon film. FIB milling was performed with a dual beam apparatus (FEI Strata DB235 M) that combines a 30 keV—Ga+ FIB with a thermal field emission scanning electron microscope, having spatial resolutions of 6 nm and 2 nm, respectively [126–128]. Particularly, the slits were produced with a 10 pA ion beam with a spot-size of about 10 nm. The beam was scanned over 100 × 1500 nm rectangular patterns, 450 nm spaced, 5 s for each pattern. The passage through the gold-carbon bilayer was monitored via the change in brightness of the ion-induced secondary electron emission. The slit width, length, and spacing are 95 nm, 1550 nm, and 450 nm, respectively. As a general rule, the TEM current intensity must be high to collect statistics within a time interval that guarantees a stable operation of the microscope, but, at the same time, the current should be very low to guarantee the necessary coherence of the electrons associated with the poor brightness of the thermionic source. Eventually, a nontrivial condition was found out so that it was possible to record the position and time of electron arrivals on the detector, in the single particle regime, with the desired accuracy and resolution.

**Figure 34:**
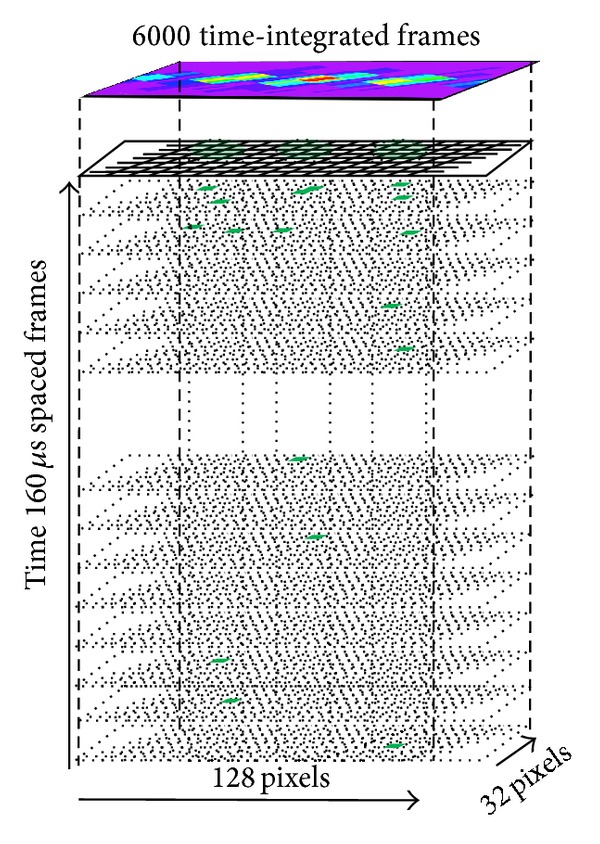
Pictorial view of the stack of frames collected in a typical two-slit interference run.

A pictorial view of the stack of frames collected in a typical run is shown in Figure 34. In particular, the frame rate has been chosen in order to contain, at the percent level, the fraction of frames with electron multiplicity higher than one. In a typical run, 131 k nonempty frames were recorded during about 5 minutes, with a frame rate of 6.25 kfps. The plot in Figure 34 reports a measurement with a BCO time of 160 *μ*s to sufficiently separate the electrons, one from each other.

**Figure 35:**
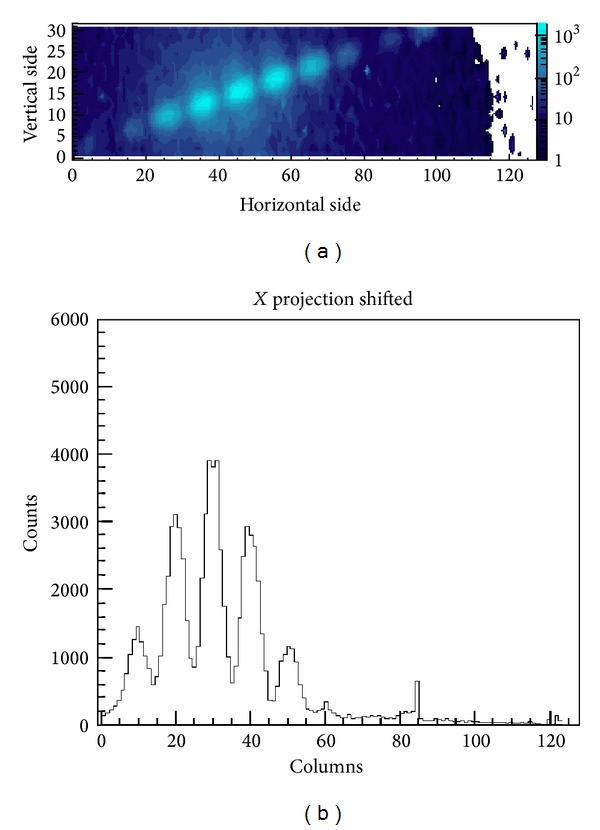
Interference scatter plot obtained by adding-up the stack of frames (a) and its projection along the vertical axis (b).

The interference scatter plot appears as expected from the interference of the two slits, placed at a certain distance, modulated by the diffraction due to their widths (Figure 32(a)). More quantitative information, like the ratio between distance and width of the slits (which does not depend on the magnification of the interference figure) or the degree of coherence can be extracted by fitting the line scan obtained by averaging the data along the vertical direction (Figure 32(b)).  Figure 35 shows that in these conditions 95.2% of the collected frames are empty, 0.1% have multiple hits, and 4.7% one-hit events. The 0 bin is not shown because we only represent the hit pixels. Moreover, by comparing the average time distance between the detected electrons, which was 3.1 ms, to the time of flight within the electron microscope, which is about 10 ns, we see that the signal of one electron is read out before the next one is emitted.

**Figure 36:**
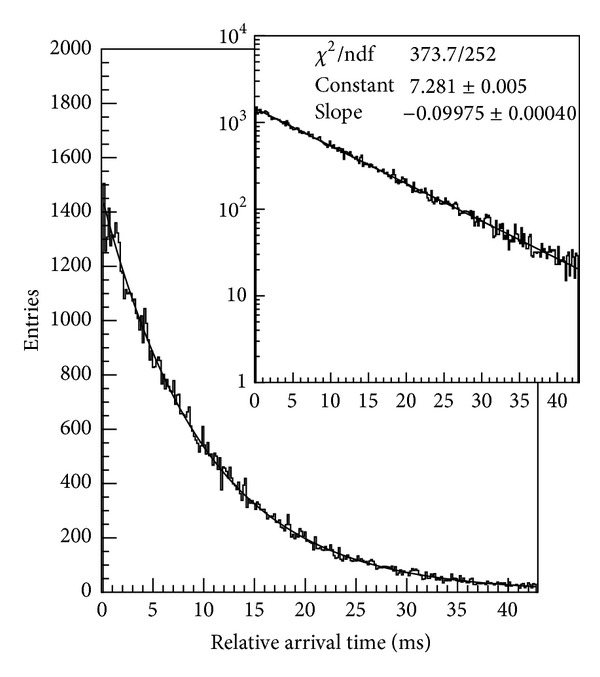
Multiplicity distribution of hit pixels within the frames.

**Figure 37:**
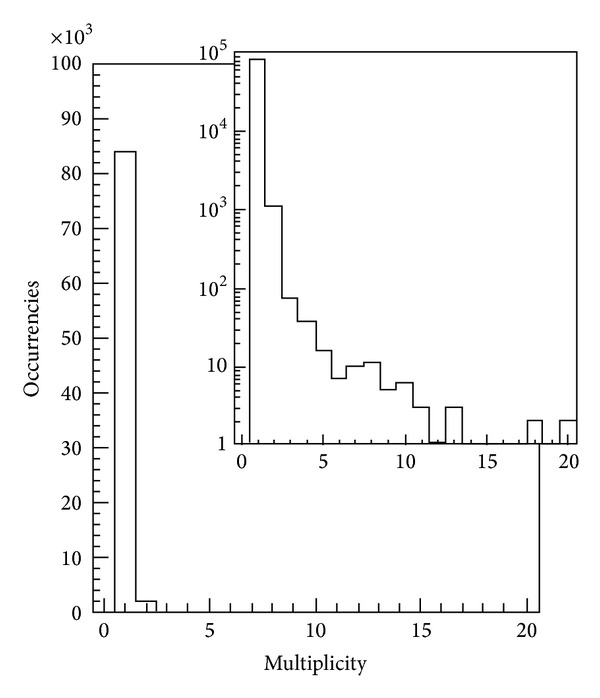
Distribution of time interval between two consecutive nonempty frames.

From the measurement of the time interval that separate two adjacent nonempty frames we get the distribution of the arrival time of the electrons on the detector, shown in Figure 37. In more detail, the exponential behavior of uncorrelated electrons is observed as expected for a Poisson distribution of events. Then, only when the different frames are added up to form a single image the typical diffraction-interference pattern of single electrons become clearly visible (see Figure 36).

A two-slit Young-Feynman experiment in the closest form to the original proposal was reproduced by coupling modern specimen preparation methods and by inserting a new pixel detector [129] in an electron microscope. The electron arrival time distribution and the buildup of interference pattern of the single electrons on the screen were measured for the first time, and this is what characterizes this work [130–134]. In fact, in the past similar experiments obtained the same pattern from single electron interference, but the electron time arrival distribution was not measured.

The high statistics of single electrons might make possible to further study the detailed properties of interference pattern formation. The system developed has an excellent potential in the field of electron microscopy, especially in the investigation of rare phenomena both in static and time-dependent regime.

In the near future we are planning to insert a micrometer positioning system within the electron microscope to create a facility for a detailed characterization of electronic sensor chips. In particular, sensor parameters such as minimum detectable energy, hardness against single event effects, and electronic noise might be easily measured using the laboratory approach here described. This should be considered as an attractive opportunity since usually these tests are performed on particle beams at the accelerators with much higher costs.

The solution features interpixel wiring independent of the size of the matrix since there are no point-to-point wires—all lines are global. This readout approach can easily be extended to any size matrix, as it is independent of the number of pixels. Here it is proposed for pixel devices that cannot be read out so easily using traditional, insufficiently fast architectures such as token-passing techniques. In addition, monolithic components that gather together matrix of pixels and readout logic would benefit from the approach given that large wire buses do not exit chips. The readout speed can be compared with HEPE detectors, rather than with the speed of HEPE detectors. For low-occupancy devices, this technique could also be used in future improvements in the electronics of physics experiments. This could match the requirements of future monolithic pixel detectors requiring robust on-chip digital sparsification and be considered for possible applications in first level triggers on tracks in vertex detectors. For this application field there are many ongoing research projects exploiting increasingly challenging microelectronics technologies and designing a larger number of sensors. It follows therefore that readout architectures must also play a significant role.

This architecture is clearly feasible as it does not place great demands on modern microelectronics technologies and does not require more sophisticated pixels compared to the ones recently used in HEPE.

Studies for fast readout for matrices of pixels are still ongoing, as they are required for applications to future colliders and follow a big effort of huge collaborations.

## 15. Summary

This document presents an overview on the latest architectures used to read out data from a matrix of pixels, in particular for high-energy physics applications (HEPE). These techniques originate from the commonly used charge-coupled devices (CCD), which are commercial off-the shelf (COTS) components. However, to fulfill the stringent HEPE requirements, the readout speed has been studied and investigated in depth over the recent years. As a consequence, the scientists designing electronics for the experiments have proposed many reasonable solutions. The always increasing demand of readout speed, of low-power, and of radiation hard architectures has lead the HEPE community to improve the more common commercial readout circuits. Today, as the HEPE collaborations are still involved in challenging upgrades [135–138] for the physics research, also the machines are pushed to obtain performances never reached until now.

New technologies can give a hand to the never-ending demand of knowledge and understanding of the outside world and should be the engine to drive new ideas to solve problems and address unsolved questions of even more complex systems.

## Figures and Tables

**Table 1:** 

Phase	RES_r	RES_c	OR_r	OR_c
Sample	1	0	*Z*	〈Pixel〉
Hold-Mask	0	0	*Z*	〈Pixel〉
Hold-Read	0	1	〈Pixel〉	〈Pixel〉
Reset	1	1	0	0

**Table 2:** 

BC clock periods (*μ*s)					RDclk (MH*z*)
99.7	99.5	99.3	99.2	99.0	**100**
99.6	99.4	99.1	99.1	98.8	**80**
97.5	98.9	98.8	98.8	98.4	**60**

**0.25**	**0.5**	**1**	**1.5**	**2**	
